# Long-Term Prognostic Value of Cognitive Impairment on Top of Frailty in Older Adults after Acute Coronary Syndrome

**DOI:** 10.3390/jcm10030444

**Published:** 2021-01-24

**Authors:** Juan Sanchis, Clara Bonanad, Sergio García-Blas, Vicent Ruiz, Agustín Fernández-Cisnal, Clara Sastre, Arancha Ruescas, Ernesto Valero, Jessika González, Anna Mollar, Gema Miñana, Julio Núñez

**Affiliations:** 1 Servicio de Cardiología, Hospital Clínico Universitario de Valencia, INCLIVA, Universidad de Valencia, CIBERCV, 46010 Valencia, Spain; clarabonanad@gmail.com (C.B.); sergiogarciablas@gmail.com (S.G.-B.); vicente.ruiz@uv.es (V.R.); fecia82@gmail.com (A.F.-C.); clarasastre90@hotmail.com (C.S.); ernestovaleropicher@hotmail.com (E.V.); jessikabeg89@gmail.com (J.G.); annam.mollar@gmail.com (A.M.); gemineta@gmail.com (G.M.); yulnunez@gmail.com (J.N.); 2 Departamento de Fisioterapia, Universidad de Valencia, 46010 Valencia, Spain; arancha.ruescas@uv.es

**Keywords:** cognitive impairment, frailty, acute coronary syndrome

## Abstract

Frailty is a marker of poor prognosis in older adults after acute coronary syndrome. We investigated whether cognitive impairment provides additional prognostic information. The study population consisted of a prospective cohort of 342 older (>65 years) adult survivors after acute coronary syndrome. Frailty (Fried score) and cognitive function (Pfeiffer’s Short Portable Mental Status Questionnaire—SPMSQ) were assessed at discharge. The endpoints were mortality or acute myocardial infarction at 8.7-year median follow-up. Patient distribution according to SPMSQ results was: no cognitive impairment (SPMSQ = 0 errors; *n* = 248, 73%), mild impairment (SPMSQ = 1–2 errors; *n* = 52, 15%), and moderate to severe impairment (SPMSQ ≥3 errors; *n* = 42, 12%). A total of 245 (72%) patients died or had an acute myocardial infarction, and 216 (63%) patients died. After adjustment for clinical data, comorbidities, and Fried score, the SPMSQ added prognostic value for death or myocardial infarction (per number of errors; HR = 1.11, 95%, CI 1.04–1.19, *p* = 0.002) and death (HR = 1.11, 95% 1.03–1.20, *p* = 0.007). An SPMSQ with ≥3 errors identified the highest risk subgroup. Geriatric conditions (SPSMQ and Fried score) explained 19% and 43% of the overall chi-square of the models for predicting death or myocardial infarction and death, respectively. Geriatric assessment after acute coronary syndrome should include both frailty and cognitive function. This is particularly important given that cognitive impairment without dementia can be subclinical and thus remain undetected.

## 1. Introduction

Geriatric conditions beyond aging influence clinical outcomes in older adults with acute coronary syndrome [1]. Frailty and comorbidities are the most extensively researched geriatric conditions [2,3]. Few studies have evaluated cognitive impairment in patients with acute coronary syndrome [4,5,6,7,8]. In a recent systematic review, the reported prevalence rates varied substantially (9–85%) [9]. On the other hand, most studies have focused mainly on short-term outcomes. With the increase in life expectancy, the prevalence of cognitive impairment is a matter of concern in patients admitted with an acute coronary syndrome, and its prognostic influence constitutes a considerable issue.

The Fried scale is probably the most frequently used frailty score with demonstrated prognostic value in cardiovascular diseases [2]. Cognitive impairment is not considered in the Fried scale and the issue of whether cognition should be included as a domain of frailty is a matter of controversy. Frailty also favors the development of cognitive impairment [10]. Indeed, the co-occurrence of frailty and cognitive impairment has been defined as “cognitive frailty” [11]. Cognitive dysfunction has been found predictive of death, independently of frailty, in non-institutionalized and community-dwelling older adults [11,12]. Yet this is an understudied area after acute coronary syndrome.

The current study involved a prospective cohort of older adults hospitalized for acute coronary syndrome. Unlike most other research in the field, both frailty and cognitive function were assessed during hospitalization and the follow-up extended to 8 years. Our main goal was to evaluate the prognostic impact of cognitive impairment on long-term outcomes.

## 2. Materials and Methods

### 2.1. Study Design

The study population consisted of a prospective cohort (1 October 2010–1 February 2012) of 342 old adults at Hospital Clínico Universitario in Valencia, Spain. A detailed description of this cohort is reported elsewhere [13,14]. In brief, inclusion criteria were survivors after acute coronary syndrome (either ST-segment elevation or non-ST-segment elevation acute coronary syndrome), age over 65 years, and written informed consent for evaluation of geriatric condition upon hospital discharge. Exclusion criteria were prior known heart disease other than coronary artery disease (such as heart valve disease or cardiomyopathy) and an indication of coronary surgery during the index hospitalization because the assessment of the geriatric conditions and cognitive function was timed at discharge, so the surgical procedure would confound their assessment. The study was reviewed and approved by the Clinical Research Ethics Committee of the University Hospital Clinic in Valencia.

Patient management was at the discretion of the attending physician. A number of variables were collected from clinical assessment during hospitalization (age, gender, coronary risk factors, prior history of ischemic heart disease, prior hospitalization for heart failure, admission heart rate and blood pressure, and Killip class), as well as electrocardiograms (ST-segment deviation, atrial fibrillation at admission), routine blood tests (high-sensitivity troponin T levels, admission hemoglobin level, and glomerular filtration rate), and echocardiograms (left ventricular ejection fraction). The GRACE score for 6-month mortality was also calculated.

### 2.2. Frailty and Cognitive Assessment

On the day before hospital discharge, frailty status was evaluated using the Fried score, which includes slowness, weakness, low physical activity, exhaustion, and shrinking (unintentional weight loss) (Table 1) [15]. The predictive value of each component of the Fried score has been published previously [16].

Cognitive function was assessed using Pfeiffer’s Short Portable Mental Status Questionnaire (SPMSQ), a brief 10-item questionnaire that has proved to be a sensitive and specific screening test for cognitive impairment (Table 2) [17,18,19,20]. Based on prior studies which found a 3-error cutoff to define moderate to severe cognitive impairment, the patient population was categorized into three subgroups: no cognitive impairment (SPMSQ = 0 errors), mild impairment (SPMSQ = 1–2 errors), and moderate to severe impairment (SPMSQ ≥ 3 errors) [18,19,20].

### 2.3. Outcomes

The endpoints were all-cause mortality and readmission for acute myocardial infarction at long-term follow-up. The follow-up period began at hospital discharge and continued until April 2020. Seven patients were lost to follow-up. We collected information on endpoints from the hospital files or outpatient department. In patients who failed to return to the hospital or outpatient department, the information was obtained by contacting the patient or the general physician.

### 2.4. Statistical Analysis

Continuous variables were expressed as median ± standard deviation and compared between the three SPMSQ categories using the ANOVA test. Categorical variables were expressed as absolute values and percentages and compared using the chi-square test. Correlation between frailty (Fried score) and cognitive function (SPMSQ) was analyzed using the Spearman coefficient. Univariable and multivariable (ordinal regression) analysis was carried out to identify independent determinants of a worse SPMSQ category.

Kaplan–Meier curves and a univariable Cox regression model were built to compare outcomes between SPMSQ categories for death or myocardial infarction and for death. The log-rank test, the hazard ratio (HR), and 95% confidence intervals (CI) were estimated. In addition, SPMSQ predictive value was estimated after adjusting for clinical data and comorbidities (full list presented in Table 1) as well as frailty status (Fried score), using univariable and multivariable Cox regression analyses (backward method). Those variables related (*p* < 0.20) to the outcomes were chosen for the multivariable analysis. SPMSQ was used as a continuous as well as a categorical variable (taking SPMSQ = 0 errors as the reference category). The Cox regression model’s proportional hazards assumption was tested with individual Schoenfeld tests and linearity of continuous variables was assessed visually with Martingale residuals plots. Finally, the relative importance of the variables included in the final Cox models (cardiac factors, comorbidities, and geriatric conditions—Fried score and SPMSQ) was computed as the proportion of the full model log-likelihood that is explainable by each predictor individually.

Statistical analysis was performed using SPSS version 26.0 software (SPSS, Inc., Chicago, IL, USA) and the rms package for R (Frank E Harrell Jr (2020); rms: Regression Modeling Strategies. R package version 6.0–1 [21].

## 3. Results

### 3.1. Patient Population Characteristics and Cognitive Function

The mean age of the cohort was 77 ± 7 years, and 196 (57%) were male. Mean SPMSQ values were 0.83 ± 1.82 errors. Patient distribution by SPMSQ category was as follows: no cognitive impairment (SPMSQ = 0 errors; *n* = 248, 73%), mild impairment (SPMSQ = 1–2 errors; *n* = 52, 15%), and moderate to severe impairment (SPMSQ ≥3 errors; *n*= 42, 12%).

Table 3 shows the clinical characteristics across SPMSQ categories. Patients with moderate to severe cognitive impairment were older, more frequently women, and presenting heart failure (Killip class ≥2) at admission and had lower hemoglobin levels and worse renal function. Overall, they showed a higher risk profile according to the GRACE score. Adherence to the guideline-recommended medication at discharge was high and without differences between SPMSQ groups. On the other hand, there was a non-significant trend to lower in-hospital revascularization rate in patients with impaired cognitive function. Percutaneous coronary intervention was used in all cases.

### 3.2. Outcomes

**Figure 1:**
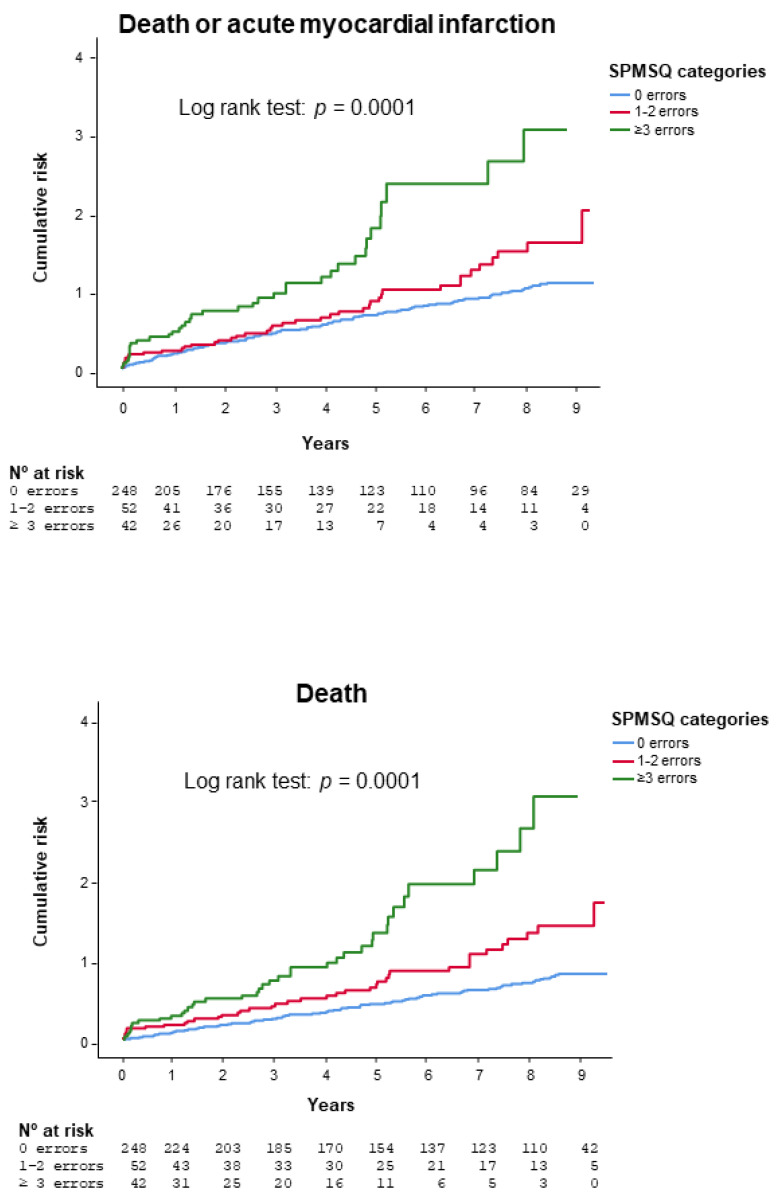
Kaplan–Meier curves comparing SPMSQ categories for death or myocardial infarction (**top**) or death (**bottom**)**.** Abbreviations: SPMSQ = Short Portable Mental Status Questionnaire.

Median follow-up was 8.7 years (interquartile interval 8.3–9.1) for the survivors and 5.6 years (interquartile interval 2.4 to 8.5) for the entire patient population. A total of 216 (63%) patients died, 78 (23%) suffered acute myocardial infarction, and 245 (72%) died or had acute myocardial infarction. Figure 1 shows the Kaplan–Meier curves comparing SPMSQ categories. Mortality or acute myocardial infarction rate increased across SPMSQ categories (SPMSQ = 0 errors, 65.3%; SPMSQ = 1–2 errors, 82.7%; SPMSQ ≥ 3 errors, 95.2%; *p* = 0.0001; log rank test). Differences were significant for 1–2 errors (HR = 1.40, 95% CI 1.0 to 1.97, *p* = 0.05) and ≥3 errors (HR = 2.56, 95% CI 1.80 to 3.63, *p*= 0.0001), taking 0 errors as the reference category. The same was observed in terms of mortality (SPMSQ = 0 errors, 54.4%; SPMSQ = 1–2 errors, 78.8%; SPMSQ ≥ 3 errors, 95.2%; *p* = 0.0001; log rank test). Again, the differences were significant for the 1–2 errors (HR = 1.76, 95% CI 1.24 to 2.51, *p* = 0.002) and ≥3 errors (HR = 3.22, 95% CI 2.26 to 4.62, *p* = 0.0001) categories. The Kaplan–Meier curves depict progressive separation from the start, with a further deviation from the 5-year follow-up onwards. The results were similar in the subgroup of patients older than 75 years (Figures S1 and S2).

**Figure 2:**
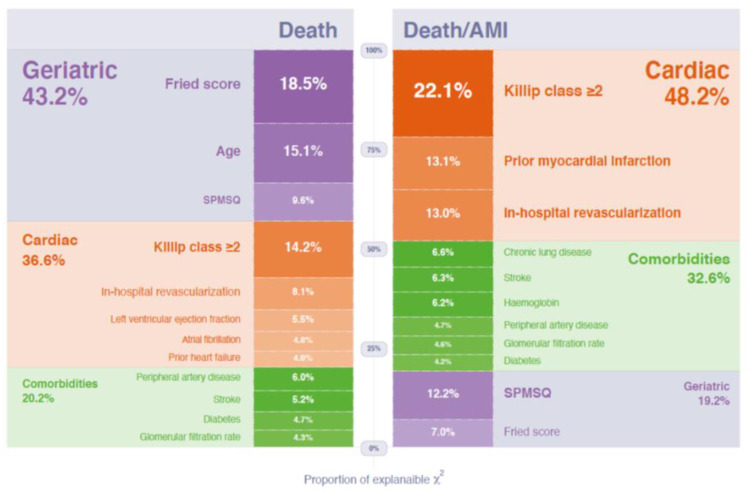
Relative importance of the variables included in the Cox models as a percentage of the overall chi-square explained by each variable, for death or myocardial infarction (top) or death (bottom). Abbreviations: AMI = acute myocardial infarction. SPMSQ = Short Portable Mental Status Questionnaire.

Table 4 shows the multivariable model for death or acute myocardial infarction. The clinical variables associated with higher risk were: prior myocardial infarction, Killip class ≥ 2 at presentation, revascularization at the index episode, and comorbidities (diabetes, prior stroke, peripheral artery disease, chronic lung disease, anemia, and renal insufficiency). Frailty was also predictive (per points of the Fried score; HR = 1.19, 95% CI 1.03 to 1.37, *p* = 0.019) and the SPMSQ added prognostic value (per number of errors; HR = 1.11, 95% CI 1.04 to 1.19, *p* = 0.002) on top of frailty, cardiac factors, and comorbidities. The relative importance of the variables included in the Cox model is also shown in Table 2. SPMSQ was the fourth most important predictor, while the Fried score was the fifth in terms of the proportion of the overall chi-square of the model. The subgroup with ≥3 errors showed the highest risk (in comparison with the subgroup with 0 errors: HR = 1.68, 95% CI 1.17 to 2.42, *p* = 0.005). There were no differences between the subgroups with 1–2 and 0 errors (HR = 1.11, 95% CI 0.78 to 1.58, *p* = 0.56). Figure 2 shows the percentage of the explainable chi-square of the model by each variable grouped as cardiac factors, comorbidities, and geriatric conditions. Cardiac factors (prior myocardial infarction, admission Killip class ≥ 2, and in-hospital revascularization) explained 48.2% of the overall chi-square of the model, comorbidities (diabetes, prior stroke, peripheral artery disease, chronic lung disease, hemoglobin, and glomerular filtration rate) explained 32.6%, and geriatric conditions (SPSM and Fried score) explained 19.2%.

Table 5 shows the multivariable model for mortality. Again, the Fried score (HR = 1.34, 95% CI 1.15 to 1.55, *p* = 0.0001) and SPMSQ (HR = 1.11, 95% 1.03 to 1.20, *p* = 0.007) were associated with mortality risk. The Fried score was the most important predictor, while SPMSQ was the fourth in terms of the proportion of the overall chi-square of the model. The differences between the ≥3 and 0 error subgroups did not reach statistical significance (*p* = 0.12). Geriatric conditions (Fried score, age, and SPSM) explained 43.2% of the chi-square of the model, cardiac factors (prior history of heart failure, admission Killip class ≥ 2, atrial fibrillation, in-hospital revascularization, and left ventricular ejection fraction) explained 36.6%, and comorbidities (diabetes, prior stroke, peripheral artery disease, and glomerular filtration rate) explained 20.2% (Figure 2).

Model diagnostics for the multivariable regressions, using individual Schoenfeld tests and Martingale residuals plots, are shown in Supplemental Tables S1 and S2 and Figures S3–S6.

## 4. Discussion

The main finding of this study was that cognitive impairment, measured with the SPMSQ test, provided independent prognostic information for long-term clinical outcomes in older adult survivors of an acute coronary syndrome. Remarkably, cognitive function was adjusted for clinical data as well as frailty status to mitigate the confounding interference of concomitant frailty. Patients who made ≥3 errors in the SPMSQ test had a particularly poor prognosis. Furthermore, frailty and SPSMQ were among the most important predictors of long-term outcome, together with cardiac factors and comorbidities. 

### 4.1. Cognitive Function in Older Adults with Acute Coronary Syndrome

The reported prevalence of cognitive impairment in patients with acute coronary syndrome varies considerably. In a recent systematic review, it ranged from 9 to 85%, ref. [9] with our results (15% mild impairment, 12% moderate to severe impairment) falling in the mid-range. The discrepancy between studies can be explained by different patient characteristics and tests used for cognition assessment. The highest prevalence has been described in heart failure cohorts, probably because heart failure brings about cognitive dysfunction [22,23].

Incidence of cognitive impairment increases with age and cardiovascular risk factors [24,25,26]. Conceivably, cerebrovascular disease could be one of the mechanisms involved in cognitive dysfunction [27], yet other studies have observed no significant differences in cardiovascular risk factor rates between patients with or without cognitive impairment [7]. We found older age, female sex, and frailty to be associated with cognitive impairment. A recent meta-analysis on community-dwelling older adults demonstrated that frailty prompts cognitive disorder [10]. A relationship between frailty, female gender, and cognitive impairment has also been suggested [28], and our results support this hypothesis.

Patients with acute myocardial infarction and cognitive impairment are often undertreated during hospitalization and participate less frequently in post-discharge rehabilitation programs [5,6,7]. This policy seems to be based on the assumption that guideline-recommended treatments bring only marginal benefit in these patients. Management of older patients with frailty or comorbidities is certainly challenging [29,30]. In our study, there were no differences in the medical treatment prescribed at discharge, but patients with cognitive impairment tended to undergo fewer revascularization procedures. Other studies have also observed that old patients with acute coronary syndrome and comorbidities less often undergo invasive management and in-hospital revascularization, although most of them might benefit from revascularization [30]. The indication of invasive management in patients with cognitive impairment is challenging. The percutaneous coronary intervention has become much simpler in recent years using the radial approach and new devices; therefore, it should not be ruled out in most patients with cognitive impairment.

### 4.2. Prognosis Impact

Cognitive impairment exerts a detrimental effect on prognosis among older community-dwelling adults [31]. This same effect was observed in patients hospitalized in the cardiology department for acute heart failure or any cardiac cause at 6 months and 1-year follow-up [32,33,34]. Likewise, cognitive impairment diagnosed during hospitalization influenced in-hospital and 1-year mortality in acute myocardial infarction patients [4,5,6,7]. Our study extended the observation period to 8 years after discharge, finding a sustained effect on long-term outcome, which is significant for these patients given the relatively low age of entry into the study (>65 years). Indeed, we found a continuous inverse association between cognitive impairment and death or recurrent myocardial infarction: the greater the cognitive dysfunction, the worse the prognosis. The subgroup with ≥3 errors in the SPMSQ had the poorest outcome. Though frailty was also predictive, the influence of cognitive impairment persisted on top of frailty. Furthermore, geriatric conditions (represented by frailty and cognitive impairment) were among the most important prognostic factors, along with comorbidities and cardiac predictive variables. The greater influence of geriatric conditions was on mortality. Frailty was the most important predictor of mortality, while cognitive impairment was more strongly associated with mortality or acute myocardial infarction. In the study of Gu et al., recurrent myocardial infarction was independently associated with cognitive decline at 1 year (8). The detrimental influence of cognitive impairment might be attributable to factors such as concomitant cerebrovascular disease, the coexistence of systemic vulnerability, or inherent difficulties with self-care [33].

### 4.3. Limitations

One limitation of this study is that the patient sample was drawn from a cardiology ward, where patients with moderate to severe cognitive impairment might be underrepresented; however, our mild or moderate to severe cognitive impairment rates were similar to others reported. Secondly, the acute illness and hospitalization process itself might play a role in impaired cognitive function, possibly confounding the evaluation at discharge [35]. Thirdly, we acknowledge that telephonic follow-up for all those patients who did not meet an endpoint in the same hospital might be inaccurate for adjudication purposes.

## 5. Conclusions

Cognitive impairment during hospitalization prognosticates worse long-term outcomes on top of frailty and other well-known clinical predictors, in older adults after acute coronary syndrome. Complete geriatric assessment for prognosis purposes should therefore include both frailty and cognitive function. Since cognitive impairment without dementia can be subclinical and thus remain undetected, our results support the cognitive assessment of older adult patients hospitalized for acute coronary syndrome.

## Figures and Tables

**Table 1:** Frailty assessment using the Fried score.

Fried Score [15] (0 to 5 Points)		Points
Weight loss	Self-reported unintentional weight loss of greater than 4.5 Kg in the preceding year	1
Physical activity	Minnesota Leisure Time Activity questionnaire. Men < 383 Kcal per week, women < 270 Kcal per week	1
Walk time	Time to walk 4.57 m: ≥7 s for height ≤ 173 cm in men or ≤ 159 cm in women, ≥6 s for height > 173 cm in men or >159 cm in women	1
Grip strength	Lowest 20% (by gender, body mass index) using a hand-held isometric dynamometer (Kg)	1
Exhaustion	Self-reported based on 2 questions from the Center for Epidemiological Studies—Depression scale: “I felt that everything I did was an effort” and “I could not get going,” with answers graded from 0 to 3. If 2 or 3 = exhaustion	1

**Table 2:** Cognitive function assessment using Pfeiffer’s Short Portable Mental Status Questionnaire (number of errors) [17.

Questions
1. What is the date today? Correct only when the month, date, and year are all correct.
2. What day of the week is it? Correct only when the day is correct.
3. What is the name of this place? Correct if any of the descriptions of the location is given. “My home,” the correct city/town, or the correct name of the hospital/institution are all acceptable.
4. What is your telephone number? Correct when the number can be verified or the subject can repeat the same number at a later time in the interview.
4a. What is your street address? Ask only if the subject does not have a telephone.
5. How old are you? Correct when the stated age corresponds to the date of birth.
6. When were you born? Correct only when the month, date, and year are correct.
7. Who is the president of your country now? Requires only the correct last name.
8. Who was president just before him? Requires only the correct last name.
9. What was your mother’s maiden name? Needs no verification; it only requires a female first name plus a last name other than the subjects.
10. Subtract 3 from 20 and keep subtracting 3 from each new number, all the way down. The entire series must be performed correctly to be scored as correct. Any error in the series—or an unwillingness to attempt the series—is scored as incorrect.

**Table 3:** Baseline patient characteristics across cognitive dysfunction categories according to the results in the SPMSQ.

	0 Errors *n* = 248	1–2 Errors *n* = 52	≥3 Errors *n* = 42	*p*
Age (years)	75.6 ± 6.4	80.3 ± 6.9	84.1 ± 6.6	0.0001
Female	90 (36%)	30 (58%)	26 (62%)	0.0001
Smoking	26 (11%)	4 (7.7%)	4 (9.5%)	0.826
Hypertension	205 (83%)	43 (83%)	35 (83%)	0.994
Hypercholesterolemia	149 (60%)	31 (60%)	20 (48%)	0.312
Diabetes	100 (40%)	27 (52%)	17 (41%)	0.297
Prior myocardial infarction	87 (35%)	14 (27%)	18 (43%)	0.268
Prior percutaneous coronary intervention	53 (21%)	5 (9.6%)	8 (19%)	0.148
Prior coronary artery bypass graft	18 (7.3%)	6 (11.5%)	3 (7.1%)	0.571
Prior admission for heart failure	31 (13%)	13 (25%)	8 (19%)	0.056
Prior stroke	26 (11%)	9 (17%)	9 (21%)	0.086
Peripheral artery disease	20 (8.1%)	7 (14%)	6 (14%)	0.270
Chronic lung disease	44 (18%)	7 (14%)	7 (17%)	0.755
Admission systolic blood pressure (mmHg)	142 ± 32	137 ± 34	146 ± 34	0.438
Admission heart rate (beats/minute)	80 ± 20	85 ± 24	86 ± 26	0.154
Admission Killip class ≥ 2	61 (25%)	20 (39%)	20 (48%)	0.003
ST-segment elevation myocardial infarction	49 (20%)	12 (23%)	10 (24%)	0.756
Left bundle branch block	16 (6.5%)	3 (5.8%)	6 (14%)	0.177
Admission atrial fibrillation	31 (13%)	6 (12%)	6 (14.3)	0.921
Troponin elevation	226 (91%)	48 (92%)	40 (95%)	0.661
Admission hemoglobin (g/dL)	12.7 ± 1.9	12.2 ± 1.8	12.0 ± 1.4	0.033
Admission glomerular filtration rate (mL/min/1.73 m²)	53 ± 14	49 ± 15	46 ± 16	0.007
Left ventricular ejection fraction	54 ± 13	54 ± 14	50 ± 14	0.094
GRACE score	132 ± 22	148 ± 27	153 ±26	0.0001
Fried score	1.8 ± 1.1	2.4 ± 0.89	2.7 ± 0.7	0.0001
In-hospital revascularization	118 (48%)	22 (42%)	121 (29%)	0.07
Aspirin	225 (91%)	43 (84%)	37 (88%	0.380
Clopidogrel	186 (77%)	38 (75%)	32 (76%)	0.981
Oral anticoagulants	30 (12%)	9 (18%)	3 (7%)	0.302
Statins	233 (94%)	48 (94%)	40 (95%)	0.948
Beta-blockers	218 (88%)	43 (84%)	36 (86%)	0.753
ACE inhibitors	216 (87%)	39 (77%)	38 (91%)	0.09

Abbreviations: SPMSQ = Short Portable Mental Status Questionnaire. ACE = angiotensin-converting enzyme. Frailty status (Fried score) worsened across Pfeiffer categories: 0 errors = 1.8 ± 1.1 points; 1–2 errors = 2.4 ± 0.89 points; ≥3 errors = 2.7 ± 0.7 points; *p* = 0.0001. Indeed, the Fried and SPMSQ scores correlated significantly (Spearman coefficient = 0.33, *p* = 0.0001). By ordinal regression, older age (*p* = 0.0001), higher Fried score (*p* = 0.014), and female sex (*p* = 0.043) were independently associated with a higher SPMSQ category.

**Table 4:** Multivariable model for death or acute myocardial infarction (total number of events = 245).

	HR	95% CI	*p*	Relative Importance (Proportion of Overall χ^2^)
Admission Killip ≥ 2	1.80	1.36 to 2.36	0.0001	0.140
Prior myocardial infarction	1.56	1.19 to 2.04	0.001	0.083
In-hospital revascularization	0.65	0.50 to 0.85	0.001	0.082
SPMSQ (errors)	1.11	1.04 to 1.19	0.002	0.078
Fried score (points)	1.19	1.03 to 1.37	0.019	0.044
Chronic pulmonary disease	1.46	1.06 to 2.02	0.022	0.042
Prior stroke	1.50	1.05 to 2.13	0.026	0.040
Admission hemoglobin (g/dL)	0.92	0.86 to 0.99	0.027	0.039
Peripheral artery disease	1.51	1.0 to 2.28	0.052	0.030
Admission glomerular filtration rate (per 5 mL/min/1.73 m²)	0.96	0.92 to 1.0	0.057	0.029
Diabetes	1.27	0.98 to 1.65	0.07	0.027

Abbreviations: HR = hazard ratio; CI = confidence interval. SPMSQ = Short Portable Mental Status Questionnaire.

**Table 5:** Multivariable model for mortality (total number of deaths = 216).

	HR	95% CI	*p*	Relative Importance (Proportion of Overall χ^2^)
Fried score (points)	1.34	1.15–1.55	0.0001	0.089
Age (years)	1.04	1.02–1.07	0.001	0.073
Admission Killip ≥ 2	1.72	1.25–2.37	0.001	0.069
SPMSQ test (errors)	1.11	1.03–1.20	0.007	0.046
In-hospital revascularization	0.69	0.52–0.92	0.012	0.039
Peripheral artery disease	1.60	1.04–2.47	0.032	0.029
Left ventricular ejection fraction at discharge (per 5%)	0.94	0.89–0.99	0.039	0.027
Prior stroke	1.47	1.01–2.12	0.044	0.025
Atrial fibrillation at admission	1.46	0.99–2.14	0.054	0.023
Diabetes	1.31	0.99–1.74	0.057	0.023
Admission glomerular filtration rate (per 5 mL/min/1.73 m²)	0.96	0.91–1.0	0.069	0.021
Prior admission for heart failure	1.39	0.96–2.0	0.080	0.019

Abbreviations: HR = hazard ratio; CI = confidence interval. SPMSQ = Short Portable Mental Status Questionnaire.

## Data Availability

The data underlying this article will be shared on reasonable request to the corresponding author.

## Supplementary Materials

The following are available online at https://www.mdpi.com/2077-0383/10/3/444/s1, Figure S1: Kaplan–Meier curves comparing SPMSQ categories for death or myocardial infarction in the subgroup of patients ≥ 75 years old, Figure S2: Kaplan–Meier curves comparing SPMSQ categories for death in the subgroup of patients ≥ 75 years old, Figure S3: Individual Schoenfeld tests for Cox multivariable model for mortality, Figure S4: Individual Schoenfeld tests for Cox multivariable model for death or acute myocardial infarction, Figure S5: Martingale residuals plots for linearity assessment of continuous variables. Cox model for mortality, Figure S6: Martingale residuals plots for linearity assessment of continuous variables. Cox model for death or acute myocardial infarction; Table S1: Tests of the proportional hazards assumption (individual Schoenfeld tests). Multivariable model for mortality, Table S2: Tests of the proportional hazards assumption (individual Schoenfeld tests). Multivariable model for death or acute myocardial infarction.

## References

[1] Singh M., Alexander K., Roger V.L., Rihal C.S., Whitson H.E., Lerman A., Jahangir A., Nair K.S. (2008). Frailty and its potential relevance to cardiovascular care. Mayo Clin. Proc..

[2] Afilalo J., Alexander K.P., Mack M.J., Maurer M.S., Green P., Allen L.A., Popma J.J., Ferrucci L., Forman D.E. (2014). Frailty assessment in the cardiovascular care of older adults. J. Am. Coll. Cardiol..

[3] Sanchis J., Soler M., Núñez J., Ruiz V., Bonanad C., Formiga F., Valero E., Martínez-Sellés M., Marín F., Ruescas A. (2019). Comorbidity assessment for mortality risk stratification in elderly patients with acute coronary syndrome. Eur. J. Intern. Med..

[4] Hovanesyan A., Rich M.W. (2008). Outcomes of acute myocardial infarction in nonagenarians. Am. J. Cardiol..

[5] Sloan F.A., Trogdon J.G., Curtis L.H., Schulman K.A. (2004). The effect of dementia on outcomes and process of care for Medicare beneficiaries admitted with acute myocardial infarction. J. Am. Geriatr. Soc..

[6] Gharacholou S.M., Reid K.J., Arnold S.V., Spertus J., Rich M.W., Pellikka P.A., Singh M., Holsinger T., Krumholz H.M., Peterson E.D. (2011). Cognitive impairment and outcomes in older adult survivors of acute myocardial infarction: Findings from the translational research investigating underlying disparities in acute myocardial infarction patients’ health status registry. Am. Heart J..

[7] Bagai A., Chen A.Y., Udell J.A., Dodson J.A., McManus D.D., Maurer M.S., Enriquez J.R., Hochman J., Goyal A., Henry T.D. (2019). Association of Cognitive Impairment With Treatment and Outcomes in Older Myocardial Infarction Patients: A Report From the NCDR Chest Pain-MI Registry. J. Am. Heart Assoc..

[8] Gu S.Z., Beska B., Chan D., Neely D., Batty J.A., Adams-Hall J., Mossop H., Qiu W., Kunadian V. (2019). Cognitive Decline in Older Patients With Non- ST Elevation Acute Coronary Syndrome. J. Am. Heart Assoc..

[9] Zhao E., Lowres N., Woolaston A., Naismith S.L., Gallagher R. (2020). Prevalence and patterns of cognitive impairment in acute coronary syndrome patients: A systematic review. Eur. J. Prev. Cardiol..

[10] Borges M.K., Canevelli M., Cesari M., Aprahamian I. (2019). Frailty as a Predictor of Cognitive Disorders: A Systematic Review and Meta-Analysis. Front. Med. (Lausanne).

[11] Esteban-Cornejo I., Cabanas-Sánchez V., Higueras-Fresnillo S., Ortega F.B., Kramer A.F., Rodriguez-Artalejo F., Martinez-Gomez D. (2019). Cognitive Frailty and Mortality in a National Cohort of Older Adults: The Role of Physical Activity. Mayo Clin. Proc..

[12] Aliberti M.J.R., Cenzer I.S., Smith A.K., Lee S.J., Yaffe K., Covinsky K.E. (2019). Assessing Risk for Adverse Outcomes in Older Adults: The Need to Include Both Physical Frailty and Cognition. J. Am. Geriatr. Soc..

[13] Sanchis J., Bonanad C., Ruiz V., Fernández J., García-Blas S., Mainar L., Ventura S., Rodríguez-Borja E., Chorro F.J., Hermenegildo C. (2014). Frailty and other geriatric conditions for risk stratification of older patients with acute coronary syndrome. Am. Heart J..

[14] Sanchis J., Ruiz V., Bonanad C., Valero E., Ruescas-Nicolau M.A., Ezzatvar Y., Sastre C., García-Blas S., Mollar A., Bertomeu-González V. (2017). Prognostic Value of Geriatric Conditions Beyond Age After Acute Coronary Syndrome. Mayo Clin. Proc..

[15] Fried L.P., Tangen C.M., Walston J., Newman A.B., Hirsch C., Gottdiener J., Seeman T., Tracy R., Kop W.J., Burke G. (2001). Frailty in older adults: Evidence for a phenotype. J. Gerontol. A Biol. Sci. Med. Sci..

[16] Sanchis J., Ruiz V., Sastre C., Bonanad C., Ruescas A., Fernández-Cisnal A., Mollar A., Valero E., Blas S.G., González J. (2020). Frailty tools for assessment of long-term prognosis after acute coronary syndrome. Mayo Clin. Proc. Innov. Qual. Outcomes.

[17] Pfeiffer E. (1975). A short portable mental status questionnaire for the assessment of organic brain deficit in elderly patients. J. Am. Geriatr. Soc..

[18] Erkinjuntti T., Sulkava R., Wikström J., Autio L. (1987). Short Portable Mental Status Questionnaire as a screening test for dementia and delirium among the elderly. J. Am. Geriatr. Soc..

[19] Fillenbaum G., Heyman A., Williams K., Prosnitz B., Burchett B. (1990). Sensitivity and specificity of standardized screens of cognitive impairment and dementia among elderly black and white community residents. J. Clin. Epidemiol..

[20] Martínez de la Iglesia J., Dueñas Herrero R., Onís Vilches M.C., Aguado Taberné C., Albert Colomer C., Luque Luque R. (2001). Cross-cultural adaptation and validation of Pfeiffer’s test (Short Portable Mental Status Questionnaire [SPMSQ]) to screen cognitive impairment in general population aged 65 or older. Med. Clin. (Barc).

[21] CRAN-Package rms. https://CRAN.R-project.org/package=rms.

[22] Vogels R.L., Scheltens P., Schroeder-Tanka J.M., Weinstein H.C. (2007). Cognitive impairment in heart failure: A systematic review of the literature. Eur. J. Heart Fail..

[23] Cameron J., Worrall-Carter L., Page K., Riegel B., Lo S.K., Stewart S. (2010). Does cognitive impairment predict poor self-care in patients with heart failure?. Eur. J. Heart Fail..

[24] Silbert B.S., Scott D.A., Evered L.A., Lewis M.S., Maruff P.T. (2007). Preexisting cognitive impairment in patients scheduled for elective coronary artery bypass graft surgery. Anesth Analg..

[25] Ganguli M., Fu B., Snitz B.E., Hughes T.F., Chang C.C. (2013). Mild cognitive impairment: Incidence and vascular risk factors in a population-based cohort. Neurology.

[26] Yaffe K., Vittinghoff E., Pletcher M.J., Hoang T.D., Launer L.J., Whitmer R.A., Coker L.H., Sidney S. (2014). Early adult to midlife cardiovascular risk factors and cognitive function. Circulation.

[27] Volonghi I., Pendlebury S.T., Welch S.J., Mehta Z., Rothwell P.M. (2013). Cognitive outcomes after acute coronary syndrome: A population based comparison with transient ischaemic attack and minor stroke. Heart.

[28] Mone P., Pansini A. (2020). Gait speed test and cognitive decline in frail women with acute myocardial infarction. Am. J. Med. Sci..

[29] Sanchis J., Sastre C., Ruescas A., Ruiz V., Valero E., Bonanad C., García-Blas S., Fernández-Cisnal A., González J., Miñana G. (2020). Randomized comparison of exercise intervention versus usual care in older adult patients with frailty after acute myocardial infarction. Am. J. Med..

[30] Sanchis J., Acuña J.M., Raposeiras S., Barrabés J.A., Cordero A., Martínez-Sellés M., Bardají A., Díez-Villanueva P., Marín F., Ruiz-Nodar J.M. (2020). Comorbidity burden and revascularization benefit in elderly patients with acute coronary syndrome. Rev. Esp. Cardiol..

[31] Strandberg T.E., Pitkala K.H., Tilvis R.S. (2009). Predictors of mortality in home-dwelling patients with cardiovascular disease aged 75 and older. J. Am. Geriatr. Soc..

[32] Sokoreli I., Pauws S.C., Steyerberg E.W., de Vries G.J., Riistama J.M., Tesanovic A., Kazmi S., Pellicori P., Cleland J.G., Clark A.L. (2018). Prognostic value of psychosocial factors for first and recurrent hospitalizations and mortality in heart failure patients: Insights from the OPERA-HF study. Eur. J. Heart Fail..

[33] Matsue Y., Kamiya K., Saito H., Saito K., Ogasahara Y., Maekawa E., Konishi M., Kitai T., Iwata K., Jujo K. (2020). Prevalence and prognostic impact of the coexistence of multiple frailty domains in elderly patients with heart failure: The FRAGILE-HF cohort study. Eur. J. Heart Fail..

[34] Yao S., Zheng P., Liang Y., Wan Y., Sun N., Luo Y., Yang J., Wang H. (2020). Predicting non-elective hospital readmission or death using a composite assessment of cognitive and physical frailty in elderly inpatients with cardiovascular disease. BMC Geriatr..

[35] Krumholz H.M. (2013). Post-hospital syndrome—An acquired, transient condition of generalized risk. N. Engl. J. Med..

