# Supplementary material for: Long-Term Prognostic Value of Cognitive Impairment on Top of Frailty in Older Adults after Acute Coronary Syndrome
Source: J Clin Med. 2021 Jan 24;10(3):444. doi: 10.3390/jcm10030444 (PMC7865569; doi:10.3390/jcm10030444)
Supplement: Supplementary file 1 [file jcm-10-00444-s001.pdf]

Supplemental Table 1. Tests of the proportional-hazards assumption (individual Schoenfeld tests). Multivariable model for mortality

	χ^2	p
**Fried score (points)**	0.1685	0.6815
**Age (years)**	3.5465	0.0597
**Admission Killip ≥2**	0.7452	0.3880
**SPMSQ test (errors)**	0.1764	0.6745
**In-hospital revascularization**	0.4605	0.4974
**Peripheral artery disease**	0.3884	0.5331
**Left ventricular ejection fraction at discharge (per 5%)**	13.8750	0.0002
**Prior stroke**	0.0563	0.8125
**Atrial fibrillation at admission**	0.8927	0.3447
**Diabetes**	0.0228	0.8800
**Admission glomerular filtration rate (per 5 mL/min/1.73 m^2)**	0.1056	0.7452
**Prior admission for heart failure**	0.2732	0.6012

Supplemental Table 2. Tests of the proportional-hazards assumption (individual Schoenfeld tests). Multivariable model for death or acute myocardial infarction.

	$\chi 2$	p
**Admission Killip ≥2**	1.67523	0.196
**Prior myocardial infarction**	1.06963	0.301
**In-hospital revascularization**	0.32420	0.569
**SPMSQ (errors)**	0.01102	0.916
**Fried score (points)**	0.52434	0.469
**Chronic pulmonary disease**	0.29594	0.586
**Prior stroke**	0.32085	0.571
**Admission haemoglobin (g/dL)**	0.00432	0.948
**Peripheral artery disease**	3.20607	0.073

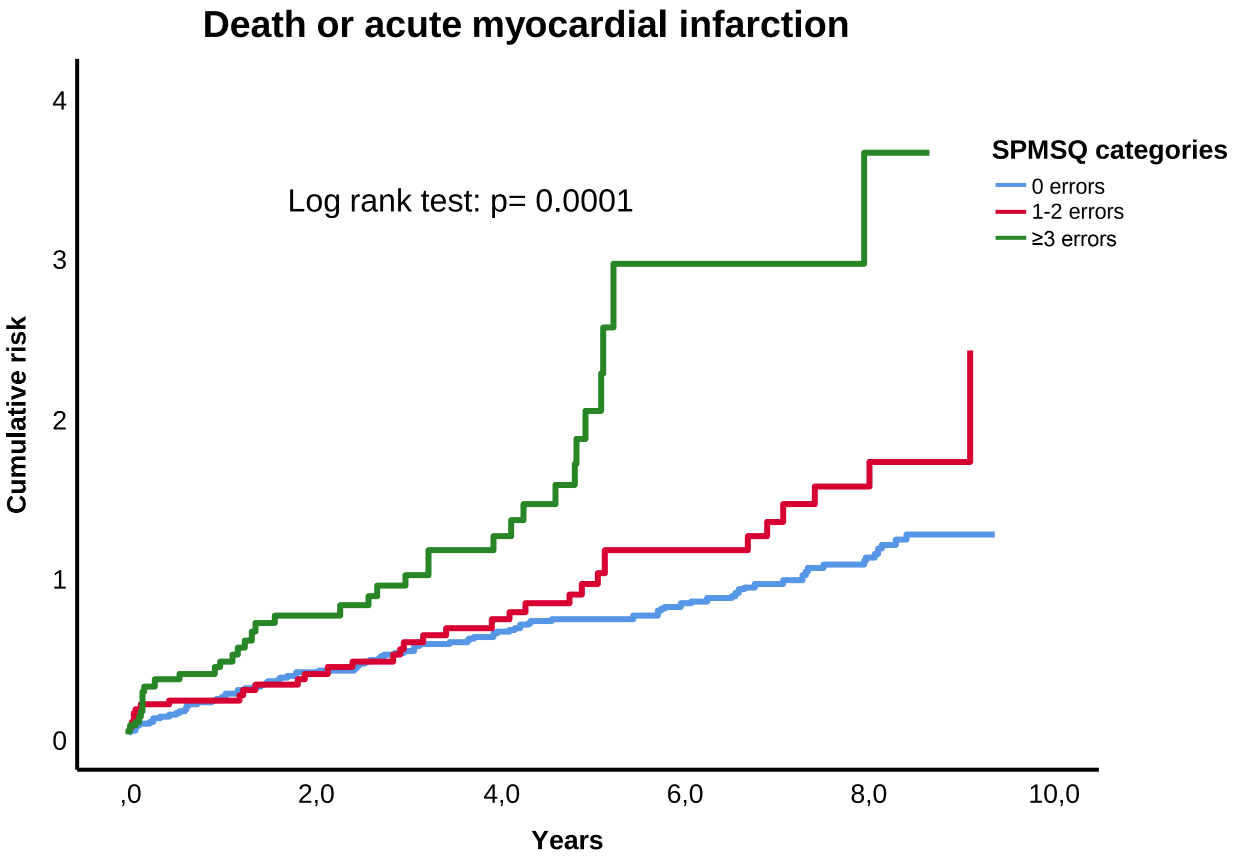

Supplemental Figure 1. Kaplan-Meier curves comparing SPMSQ categories for death or myocardial infarction in the subgroup of patients ≥75 years old**.** Differences were significant for ≥3 errors (HR= 2.52, 95% CI 1.71 to 3.72, p= 0.0001) and there was a non-significant trend for 1-2 errors (HR= 1.37, 95% CI 0.92 to 2.02, p = 0.1), taking 0 errors as the reference category. Abbreviations: SPMSQ = Short Portable Mental Status Questionnaire

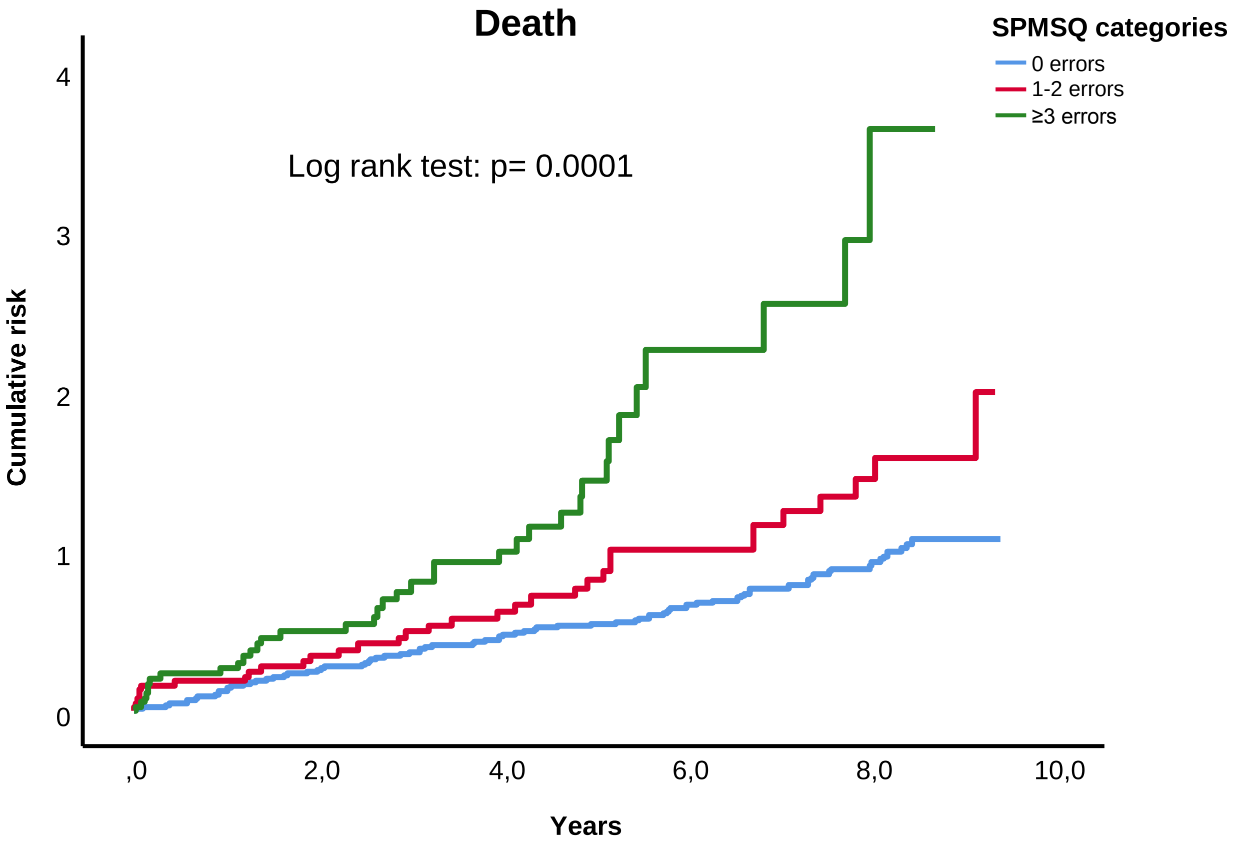

Supplemental Figure 2. Kaplan-Meier curves comparing SPMSQ categories for death in the subgroup of patients ≥75 years old. Differences were significant for 1-2 errors (HR= 1.57, 95% CI 1.05 to 2.34, p = 0.03) and ≥3 errors (HR= 2.81, 95% CI 1.89 to 4.16, p= 0.0001), taking 0 errors as the reference category. Abbreviations: SPMSQ = Short Portable Mental Status Questionnaire

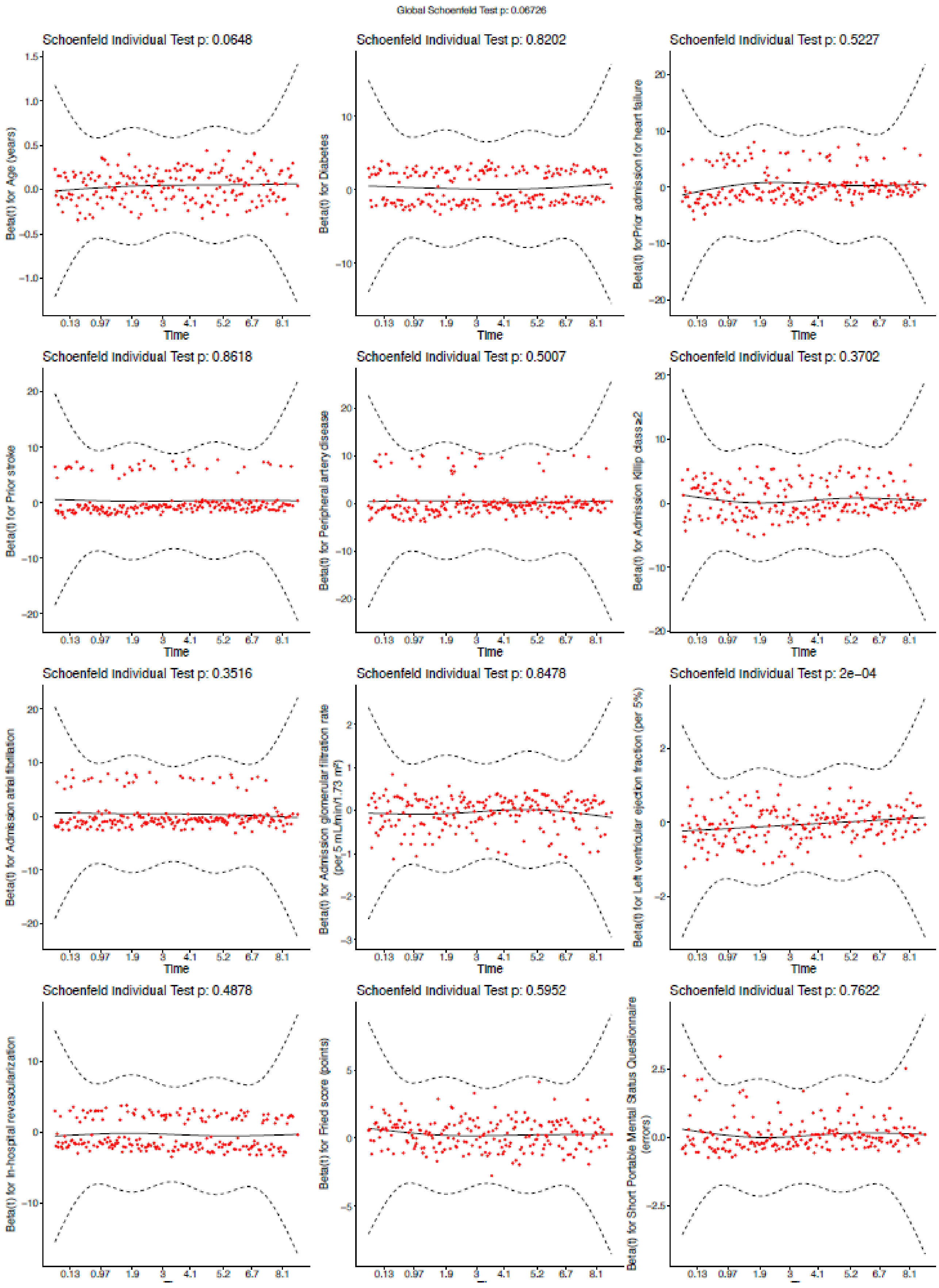

Supplemental Figure 3.. Individual Schoenfeld tests for Cox multivariable model for mortality

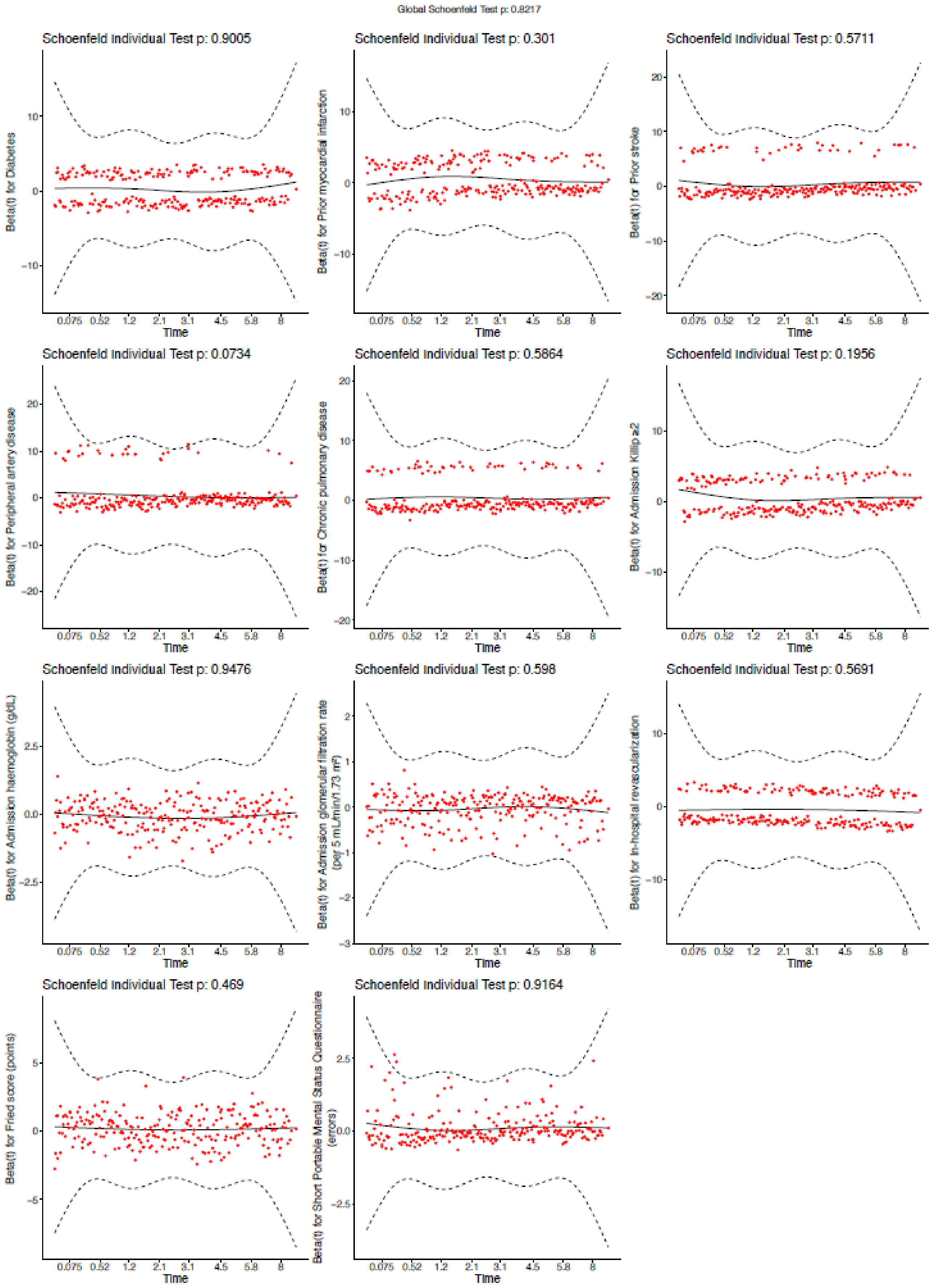

Supplemental Figure 4. Individual Schoenfeld tests for Cox multivariable model for death or acute myocardial infarction.

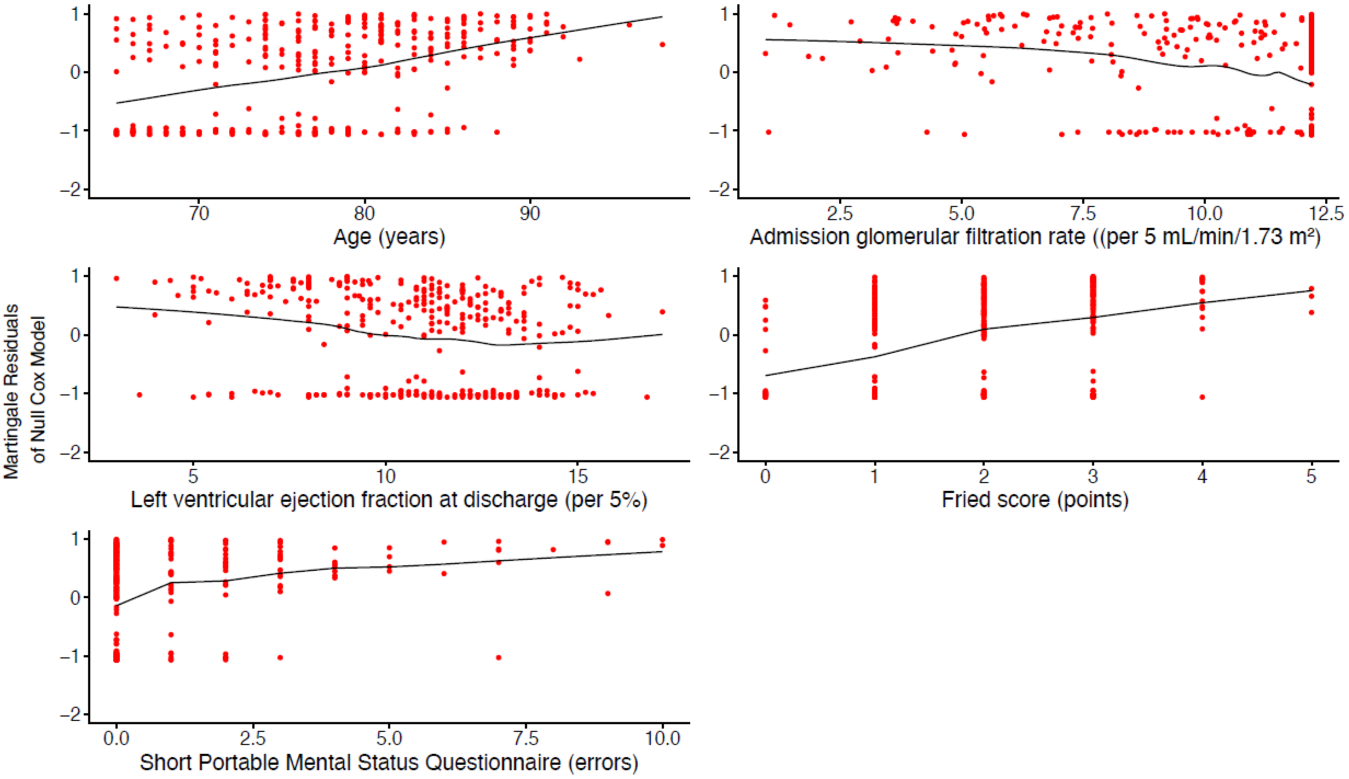

Supplemental Figure 5. Martingale residuals plots for linearity assessment of continuous variables. Cox model for mortality

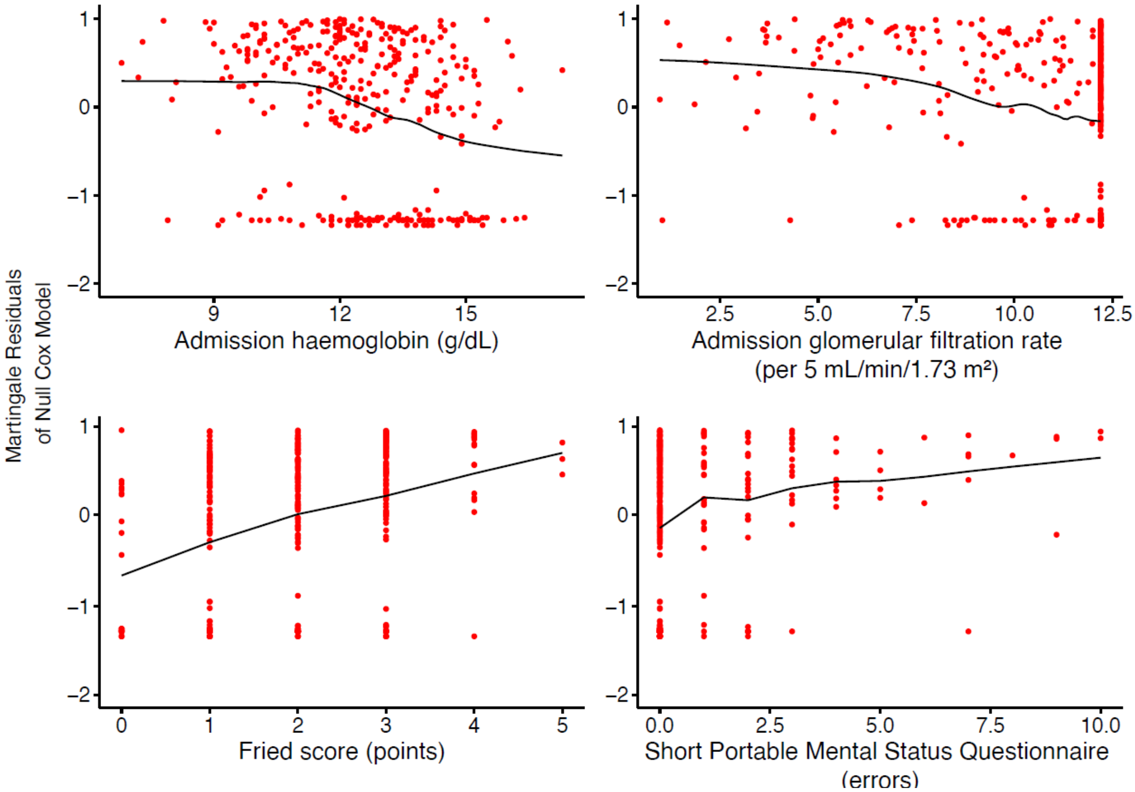

Supplemental Figure 6. Martingale residuals plots for linearity assessment of continuous variables. Cox model for death or acute myocardial infarction.
